# From New York

**Published:** 1892-11

**Authors:** P. R. Cortelyou

**Affiliations:** New York


### *CORRESPONDENCE.*

### FROM NEW YORK.

NEW YORK, October 14, 1892.

Last weeek I attended a clinic at the Methodist Episcopal Hospital in Brooklyn (known as the Seney Hospital). This is one of the most complete buildings of its kind in all its appointments, of any it has been my privilege to visit. Dr. Lewis Pilcher, who is surgeon in chief had much of the work to plan, used every opportunity in his power to have every part of the building as perfect as possible. The doctor, by the way, is one of the most eminent surgeons of Brooklyn, and a very strong advocate of antiseptic precautions during preparatory treatment; and during the operation, to the very minutest detail, everything must be antiseptic. Visitors are obliged to wear linen coats over their clothing while in the operating room, and all the trained nurses are obliged to have their heads covered with antiseptic cloths. The operating room is very complete in all its arrangements ; the table is of metal sloping from the edge to a central groove, which carries off all the fluids used in washing out the wounds. Before any operation takes place the floor of the room is scrubbed with antiseptic solution, also the table and the instruments are all sterilized, and kept in hot dishes, being so hot when handed to the surgeon, that sometimes it is hard to hold them and he is obliged to let them cool.

The first operation was made on a boy aged seven years, for partial anchylosis of the left elbow joint, resulting from a fracture of the inner condyle, which had been treated in a straight splint, and had been drawn forward by the action of the muscles, and had united in that portion, thus preventing flexion of the elbow. He made an incision about four inches long, over the inner surface of the joint ; then with a chisel removed the displaced condyle ; washed out the wound with a bichloride solution, sewed up the wound with silver wire sutures, flexed the forearm to its full extent, and put it up in plaster of paris splint, to remain for one to two weeks ; then to be taken down, and have passive motion made.

The next case was a woman, aged seventy years, with a large sarcomatous tumor of the first phalanx of the little finger of the left hand. This was removed by cutting away all of the hand but the index finger and their metacarpal bones. The wound was thoroughly washed, and the edges brought together with silk worm gut sutures ; then covered with iodoform gauze and cotton.

The third case was one of chronic synovitis of the knee joint in a woman, aged forty-five years. There was a sinus extending into the joint, the cartilages were eroded, and the synovial membrane in a gelatinous condition. He opened the joint, by making an oval incision, from the outer end of the head of the fibula over the patella, and extending to the head of the tibia. The flap was then turned back, and the synovial sac dissected off, the eroded ends of bones scraped, the track of the sinus opened and freely scraped; also all pockets which had been made by the discharge in the surrounding tissue were scraped and cleaned of all septic matter, the whole wound washed with bichloride solution, and two large drainage tubes passed through the joint. The limb was then straightened, the wound sewed up with silk worm gut sutures, and plaster dressing applied. One week after these operations they were opened and redressed, and all doing well. These cases certainly speak well for antiseptic treatment carefully carried out.

P. R. CORTELYOU, M. D.

